# Modulation Recognition of Communication Signals Based on Multimodal Feature Fusion

**DOI:** 10.3390/s22176539

**Published:** 2022-08-30

**Authors:** Xinliang Zhang, Tianyun Li, Pei Gong, Renwei Liu, Xiong Zha

**Affiliations:** School of Information Systems Engineering, PLA Strategic Support Force Information Engineering University, Zhengzhou 450001, China

**Keywords:** modulation recognition, feature fusion, RSBU-CW, PNN, multipath fading channels

## Abstract

Modulation recognition is the indispensable part of signal interception analysis, which has always been the research hotspot in the field of radio communication. With the increasing complexity of the electromagnetic spectrum environment, interference in signal propagation becomes more and more serious. This paper proposes a modulation recognition scheme based on multimodal feature fusion, which attempts to improve the performance of modulation recognition under different channels. Firstly, different time- and frequency-domain features are extracted as the network input in the signal preprocessing stage. The residual shrinkage building unit with channel-wise thresholds (RSBU-CW) was used to construct deep convolutional neural networks to extract spatial features, which interact with time features extracted by LSTM in pairs to increase the diversity of the features. Finally, the PNN model was adapted to make the features extracted from the network cross-fused to enhance the complementarity between features. The simulation results indicated that the proposed scheme has better recognition performance than the existing feature fusion schemes, and it can also achieve good recognition performance in multipath fading channels. The test results of the public dataset, RadioML2018.01A, showed that recognition accuracy exceeds 95% when the signal-to-noise ratio (SNR) reaches 8dB.

## 1. Introduction

Modulation recognition mainly refers to analyzing the noncooperative received signals through a series of processes to acquire their modulation types. How to automatically recognize the modulation types of signals quickly and accurately plays a key role in the subsequent demodulation and analysis [1].

Since the publication of the first article on modulation recognition in 1969 [2], the research on modulation recognition has been rather mature, which is mainly divided into recognition schemes based on maximum likelihood theory, recognition schemes based on feature extraction, and recognition schemes based on deep learning [3]. The recognition schemes based on the maximum likelihood theory was developed earlier, and its basic idea is that, according to the statistical characteristics of signals and to minimize the loss function as the goal, the log-likelihood function of signals is obtained through theoretical derivation and calculation, and then the appropriate threshold is selected to compare the original signal with its log-likelihood function to obtain the predicted classification results [4]. In the noncooperative communication condition, the received signals contain many unknown parameters. Recognition schemes based on maximum likelihood theory can get the theoretical optimal solution, but they need a lot of prior knowledge, have high computational complexity, and poor generalization ability. Recognition schemes based on feature extraction transform the received signals into other domain features to better characterize the modulation types. The common features include instantaneous amplitude, phase- and frequency-features, high-order cumulant, high-order cumulant spectrum, and other high-order statistics; time-frequency features, such as the short-time Fourier transform and the wavelet transform; cyclic stationary features, such as the cyclic spectral density function and the cyclic spectral correlation function. The instantaneous amplitude, phase, and frequency features have poor anti-noise ability, and the effect is not good when used alone [5]. High-order statistics can effectively suppress the interference of Gaussian noise by taking advantage of the property that the high second-order cumulant of Gaussian noise is equal to zero [6]. It is suitable for amplitude or phase modulation signals, but it needs to extract the symbol sequence synchronously. Time-frequency analysis converts one-dimensional time-domain signals into two-dimensional domain features, which can describe energy changes of signals at different times and frequencies [7]. Due to the periodic changes of signals, modulation signals have cyclic stationary characteristics, which can be characterized by the cyclic spectral density function and the cyclic spectral correlation function, but they are not suitable for short-burst signals [8].

Recognition schemes based on deep learning are currently the most popular research direction. O’Shea et al. [9] constructed the RadioML 2016.04C dataset containing 11 kinds of modulation types using GNU Radio, and in-phase and orthogonal (I/Q) components of the received signals are input into the convolutional neural network (CNN) for classification, which proves that the classification effect of the CNN is far better than traditional artificial feature extraction methods, and opens a big curtain of applications of deep learning in modulation recognition. Subsequently, O’Shea et al. [10] constructed the RadioML 2018.01A dataset containing 24 kinds of modulation types, and ResNets were used to build the recognition network to further verify the great prospect of practical applications of deep learning. Then, researchers are committed to modifying the inputs, structures, and loss functions of network models to improve the performance of modulation recognition [11,12,13]. Most modulation recognition schemes based on deep learning mainly select a single signal feature with high discrimination as the network input or optimize the network structure to extract more abstract features to improve the performance of the modulation recognition, ignoring complementarity between features in different transform domains and among different classifiers. In order to obtain better recognition performance, multimodal feature fusion technology is applied to modulation recognition. In [14], a multiscale convolutional neural network (MSN) is proposed to extract and integrate multiscale features directly from the raw I/Q signals to improve the recognition ability and robustness of the model. In [15], a waveform-spectrum multimodal fusion (WSMF) method is proposed, and Resnet is used to extract features of I/Q waveform, modulus and phase, as well as welch spectrum, square spectrum, and fourth power spectrum. The three features are flattened and spliced to make the model learn more discriminative features, so as to improve the performance. In [16], CNN-LSTM is adopted to extract temporal and spatial feature information of the I/Q waveform, modulus and phase of the original signals, and features are paired with each other to increase the diversity of features and improve the performance.

In this paper, a modulation recognition scheme based on multimodal feature fusion is proposed, which can enhance the performance of modulation recognition under different channels. Different from the existing feature fusion schemes, the contributions of the proposed scheme are as follows:

(i) From the perspective of the time-frequency domain, I/Q waveform, modulus and phase, as well as the welch spectrum, square spectrum, and fourth power spectrum are extracted as network input.
(ii) RSBU-CW12 is designed to extract high-dimensional features in space, LSTM is used to extract temporal features, and outer product operation is utilized to conduct pairwise interaction between the above-extracted spatial and temporal features.
(iii) Product-based neural networks (PNN) are adopted to enhance the ability to learn cross-features.

The rest of the paper is organized as follows. Section 2 introduces the signal model. Section 3 describes the proposed scheme, including the network structure and feature-fusion methods. Analysis of the simulation results and validation of the public dataset, RadioML 2018.01A, are shown in Section 4. Finally, a brief conclusion is given in Section 5.

## 2. Signal Model

The baseband received signal can be expressed as:

(1) y(t)=x(t)∗h(t)+n(t)

where x(t) represents the baseband transmission signal, n(t) represents the Gaussian white noise, h(t) represents the channel impulse response.

If received signals are only interfered by Gaussian white noise, h(t)=1; If there exist multiple propagation paths, such as direct beam, reflection, and refraction, the channel model can be expressed as:

(2) $$h(t)=\sum_{i=1}^{L}\alpha_{i}(t)e^{-j2\pi f_{c}\tau_{i}(t)}\delta [\tau -\tau_{i}(t)]$$

where L represents the number of discrete multipath channels, $\alpha_{i}(t)$ represents the attenuation factor of the received signals on the ith propagation path, $\tau_{i}(t)$ represents the propagation delay of the received signals on the ith propagation path.

Substituting Equation (1) into Equation (2), we can get:

(3) $$y(t)=\sum_{i=1}^{L}\alpha_{i}(t)e^{-j\theta_{i}(t)}x[\tau -\tau_{i}(t)]+n(t)$$

where $\theta_{i}(t)=2\pi f_{c}\tau_{i}(t)$.

According to Euler’s formula, the instantaneous envelope a(t) and phase θ(t) of the received signal can be expressed as follows:

(4) $$a(t)=\sqrt{{\left[\sum_{i=1}^{L}\alpha_{i}(t)x[\tau -\tau_{i}(t)]\cos\theta_{i}(t)\right]}^{2}+{\left[\sum_{i=1}^{L}\alpha_{i}(t)x[\tau -\tau_{i}(t)]\sin\theta_{i}(t)\right]}^{2}}$$

(5) $$\theta (t)=\arctan\left[-\sum_{i=1}^{L}\alpha_{i}(t)x[\tau -\tau_{i}(t)]\sin\theta_{i}(t)/\sum_{i=1}^{L}\alpha_{i}(t)x[\tau -\tau_{i}(t)]\cos\theta_{i}(t)\right]$$

The received signal is further simplified as:

(6) $$\begin{array}{l} y(t)=\sum_{i=1}^{L}\alpha_{i}(t)x[\tau -\tau_{i}(t)]\cos\theta_{i}(t)-j\sum_{i=1}^{L}\alpha_{i}(t)x[\tau -\tau_{i}(t)]\sin\theta_{i}(t)+n(t)\\ \;=a(t)e^{-j\theta (t)}+n(t) \end{array}$$

Therefore, the received signal propagated over the multipath fading channel can be regarded as numerous time-varying vectors of amplitude and phase. If the channel is a Rayleigh fading channel, the envelope of the channel response at any time follows a Rayleigh distribution, and the phase in the range (0,2π) follows a uniform distribution [17]. The corresponding probability density functions are:

(7) $$f(a)=\frac{a}{\sigma^{2}}\exp(-\frac{a^{2}}{2\sigma^{2}})$$

(8) $$f(\theta )=\left\{\begin{array}{l} \frac{1}{2\pi },\theta \in \left(0,2\pi \right)\\ 0,\;\;\;otherwise \end{array}\right.$$

where $\sigma^{2}$ represents the average power of the signal.

If the channel is a Rician fading channel, it can be viewed as the sum of direct signal and multipath signal components following a Rayleigh distribution [18]. The probability density function of the signal response can be expressed as:

(9) $$f(a)=\frac{a}{\sigma^{2}}I_{0}(\frac{Aa}{\sigma^{2}})\exp(-\frac{A^{2}+a^{2}}{2\sigma^{2}})$$

where *A* represents the amplitude of the direct signal, *I*_0_ represents the modified order 0 of the first kind of the Bessel function.

Next, the influence of channel parameters on the received signal is analyzed [19]. The coherent bandwidth of the channel can be expressed as:

(10) $$W_{c}\approx \frac{1}{T_{d}}$$

where *T_d_* represents multipath delay.

If the signal bandwidth is much larger than the coherence bandwidth, the amplitude of some frequency components of the received signal will be enhanced, and the amplitude of some frequency components will be decreased, and frequency selective fading will occur. If the signal bandwidth is much smaller than the coherence bandwidth, all frequency components of the received signal are subject to the same fading and the signal only experiences flat fading.

The coherence time of the signal can be expressed as:

(11) $$T_{c}\approx \frac{1}{f_{doppler}}$$

where *f_doppler_* represents the doppler frequency shift.

If the signal symbol period is much smaller than the channel coherence time, channel changes are slower than signal changes, and interference caused by frequency shift is not obvious and slow fading occurs. If the signal symbol period is much larger than the channel coherence time, channel changes are faster than signal changes, and adjacent frequency components interfere with each other and fast fading will occur.

## 3. The Proposed Scheme

**Figure 1:**
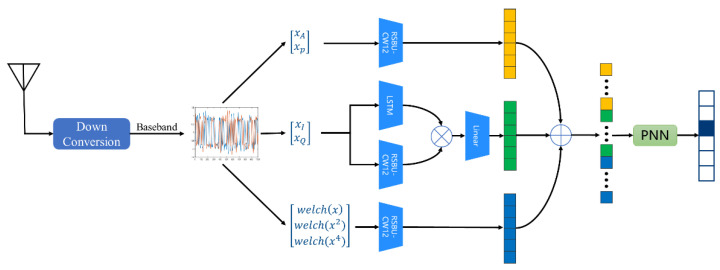
Principle framework of the proposed scheme.

Multimodal technology has been widely used in modulation recognition. However, at present, it either relies on the network to extract multi-scale feature maps [14], or the simple concatenation of transformation domain features [15], or the interaction of spatial-temporal features [16]. The work of feature fusion deserves further exploration, so this paper proposes a modulation recognition scheme based on multimodal feature-fusion to improve the performance of modulation recognition under different channel interference, whose framework is shown in Figure 1.

Firstly, the multiple transformation domain can provide multimodal information, so the I/Q waveform, modulus and phase of the received signals, as well as welch spectrum, square spectrum, and fourth power spectrum are extracted from the perspective of the time-frequency domain as network input [15]. Then, we consider the way the networks learn and incorporate multimodal features. RSBU-CW12 is designed to extract spatial features of signals. Inspired by [16], the raw I/Q signals are fed into the LSTM and the RSBU-CW12 to extract the temporal and spatial features of the signals, and then the outer product operation is performed to increase the diversity of features. Since the outer product can bring feature-dimension expansion, a fully connected layer is used to reduce the feature dimension. The modulus, phase, and spectrum features are fed into the RSBU-CW12 to extract their respective features. For the three groups of features extracted from the I/Q waveform, modulus and phase, as well as the welch spectrum, square spectrum, and fourth power spectrum, the method of direct concatenation to the full connection layer cannot achieve the full fusion of features. We adopted the PNN model to carry out feature cross-fusion for the features extracted from the network, so that the model can capture more key information.

### 3.1. Network Model Structure

**Figure 2:**
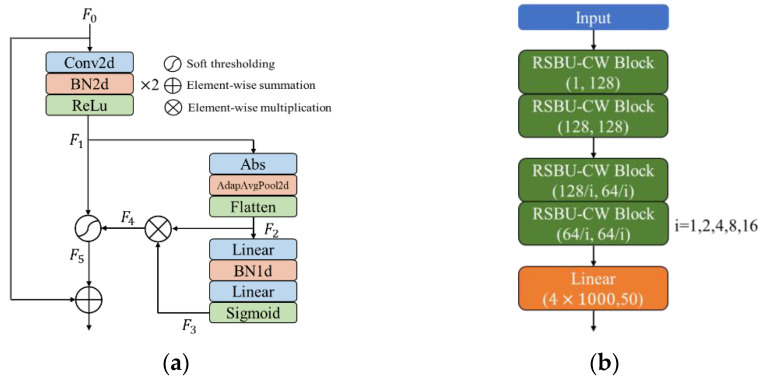
Deep residual shrinkage network model. (**a**) RSBU-CW Block; (**b**) RSBU-CW12.

To better extract signal features, a deep residual shrinkage network, RSBU-CW12, is designed, as shown in Figure 2. The RSBU-CW Block is introduced into the convolutional layer of the network, and its processing flow is mainly as follows: the initial feature input *F*_0_ is convolved twice to get the feature vector *F*_1_, and then *F*_1_ is fed into the sub neural network with soft thresholding. First, *F*_1_ takes the absolute value, and adaptive pooling and flattening are carried out to obtain one-dimensional features, *F*_2_; *F*_2_ passes through two fully connected layers and performs a sigmoid operation to get *F*_3_; *F*_4_ can be obtained by multiplying *F*_2_ and *F*_3_; redundant features are eliminated to obtain feature *F*_5_ by soft thresholding results obtained by *F*_4_ and *F*_1_; the initial input *F*_0_ and soft thresholding result *F*_5_ are added to obtain the final output result through identity mapping, as shown in Figure 2a. Finally, features extracted from the residual shrinkage module are reduced through the fully connected layer to obtain a feature vector of size 1 × 50. Traditional image network models generally employ a convolution kernel of 3 × 3, but since the network input of RSBU-CW12 is 2 × 1000 signal waveform, a convolution kernel of 1 × 3 and 2 × 3 are adopted. To make the network fully learn how to hop information between symbol sequences, the pooling layer is canceled after the convolution operation. As the number of network layers increases, a zero-padding operation is carried out before each convolution, and the step of the convolution is set to one to ensure that deep network input has enough feature information [20]. The batch normalization (BN) layer and dropout layer are also utilized to suppress overfitting.

Soft thresholding is the nonlinear transformation, and it sets features whose absolute value is less than the threshold directly to zero, and “shrinks” features whose absolute value is greater than the threshold by subtracting the threshold from them. Setting the thresholds is automatically adjusted through network training. The formula of soft thresholding and its derivative can be defined as:

(12) $$y=\left\{\begin{array}{l} x-\tau ,x>\tau \\ 0,-\tau \leq x\leq \tau \\ x+\tau ,x<-\tau \end{array}\right.$$

(13) $$\frac{\partial y}{\partial x}=\left\{\begin{array}{l} 1,\;\;\;\;\;\;x>\tau \\ 0,-\tau \leq x\leq \tau \\ 1,\;\;\;\;\;\;x<-\tau \end{array}\right.$$

where x represents the feature input, y represents the feature output, and τ represents the threshold.

### 3.2. Multimodal Feature Fusion

The proposed scheme performs feature-fusion from the following three aspects.

#### 3.2.1. Multimodal Feature Input in the Time-Frequency Domain

**Figure 3:**
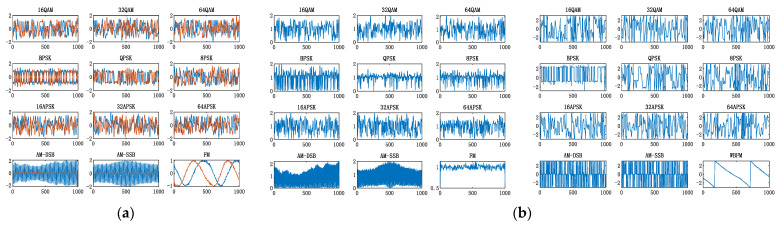

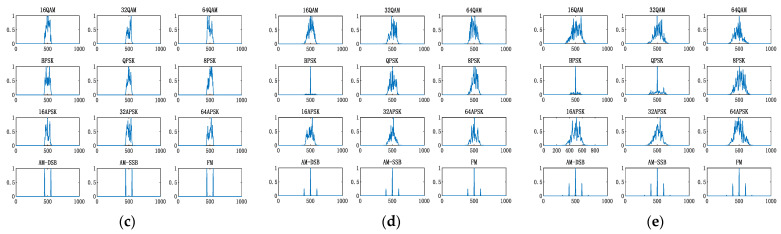
Time-frequency domain feature inputs. (**a**) I/Q waveform; (**b**) modulus and phase; (**c**) welch spectrum; (**d**) square spectrum; (**e**) fourth power spectrum.

In the signal preprocessing stage, different domain transformation features of the received signals are extracted from the perspective of the time-frequency domain. The I/Q waveform, modulus and phase, welch spectrum, square spectrum, and fourth power spectrum are taken as network inputs. Figure 3 shows the time-frequency domain feature inputs of 12 kinds of modulation types when SNR = 18 dB.

#### 3.2.2. Temporal and Spatial Feature-Fusion

For the I/Q waveform of the received signals, RSBU-CW12 is used to extract high-dimensional spatial features and get feature vectors *f_a_* of size 1 × 50; Meanwhile, LSTM is used to extract temporal features and get feature vectors *f_b_* of size 1 × 50. To fully integrate temporal and spatial features, the outer product operation is utilized to conduct pairwise interaction between the extracted two groups of features and obtain feature vectors *f_c_* of size 50 × 50. Finally, feature vector *f_c_* is reshaped to 1 × 2500, and then its dimension is reduced to 1 × 50 with fully connected layers.

#### 3.2.3. PNN Feature Cross Fusion

**Figure 4:**
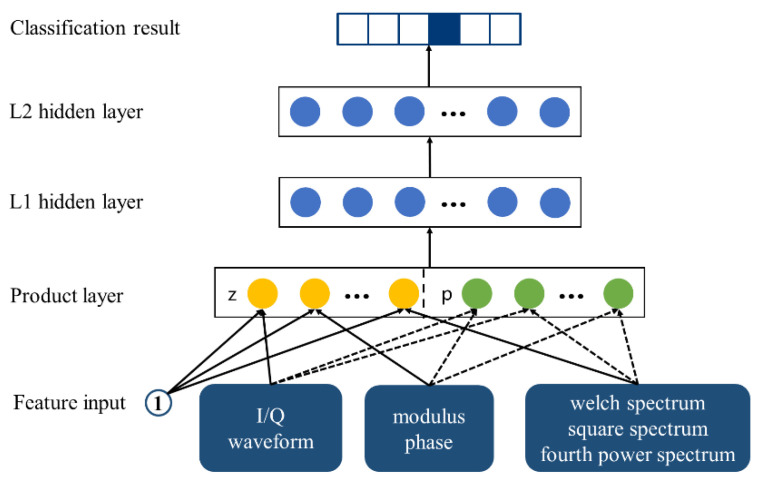
PNN model.

The proposed scheme extracts three groups of 1 × 50 feature vectors from the I/Q waveform, modulus and phase, as well as from the welch spectrum, square spectrum, and fourth power spectrum. They are stacked to obtain 3 × 50 feature vectors. The PNN model is employed to replace the fully connected layer for recognition. The PNN model mainly adds the vector product layer between feature inputs and fully a connected layer to improve the ability of learning cross-features, as shown in Figure 4.

The structure of the PNN model is mainly divided into the following parts:

(1) Features Input

The constant number “1” represents the bias, and feature input is the feature vector of size 3 × 50 extracted with a neural network, which can be defined as:

(14) $$f_{input}=\left(\begin{array}{l} f_{1}\\ f_{2}\\ f_{3} \end{array}\right)=\left(\begin{array}{l} \left[f_{11},f_{12},\ldots ,f_{1M}\right]\\ \left[f_{21},f_{22},\ldots ,f_{2M}\right]\\ \left[f_{31},f_{32},\ldots ,f_{3M}\right] \end{array}\right)$$

where *M* = 50, *f*_1_, *f*_2_, and *f*_3_ represent the feature vectors of the I/Q waveform, modulus and phase, as well as the welch spectrum, square spectrum, and fourth power spectrum extracted with a neural network, respectively.

(2) Product Layer

*f_input_* is fed into the product layer to get the linear eigenvector *f_z_*, and the nonlinear eigenvector *f_p_*. *f_z_* can be defined as:

(15) $$z=f_{input}$$

(16) $$f_{z}^{n}=W_{z}^{n}⊙z=\sum_{i=1}^{N}\sum_{j=1}^{M}{\left(W_{z}^{n}\right)}_{ij}z_{ij}$$

where *N* = 3 and $W_{z}^{n}$ represents the weight of the linear part.

Feature interaction adopts the inner product operation, *f_p_* can be defined as:

(17) $$W_{p}^{n}=\theta^{n}\theta^{nT}$$

(18) $$p=\langle f_{i},f_{j}\rangle$$

(19) $$\begin{array}{l} f_{p}^{n}=W_{p}^{n}⊙p=\sum_{i=1}^{N}\sum_{i=1}^{N}{\left(W_{p}^{n}\right)}_{ij}p_{ij}\\ \;=\sum_{i=1}^{N}\sum_{j=1}^{N}\theta_{i}^{n}\theta_{j}^{n}\langle f_{i},f_{j}\rangle =\langle \sum_{i=1}^{N}\delta_{i}^{n},\sum_{i=1}^{N}\delta_{j}^{n}\rangle \end{array}$$

where $\delta_{i}^{n}=\theta_{i}^{n}f_{i}$, $W_{p}^{n}$ represents the weight of the nonlinear part, i=1,2,…,N; j=1,2,…,N.

(3) L1 Hidden Layer

(20) $$l_{1}=\mathrm{relu}(f_{z}+f_{p}+b_{1})$$

where relu(*x*) is the linear rectification function, which can be defined as: relu(*x*) = max(0,*x*). *b*_1_ represents bias.

(4) L2 Hidden Layer

(21) $$l_{2}=\mathrm{relu}(W_{2}l_{1}+b_{2})$$

where *W*_2_ represents the weight coefficient and *b*_2_ represents bias.

## 4. Experimental Results Analysis

In this section, the performance of the multimodal feature-fusion scheme is evaluated. The generated simulation datasets of three different channels, namely Gaussian white noise, Rayleigh fading, and Rician fading, are used to verify the effectiveness of the scheme together with the public dataset RadioML2018.01A.

Experimental platform: GPU is NVIDIA TITAN Xp; CPU is Intel(R) Xeon(R) CPU E5-2650 v4 @ 2.20GHZ; memory is 256GB; deep learning framework is PyTorch 1.8.0.

Network training parameters: Training times *N_epoch_* = 50, batch size *N_batch_* = 64; the optimizer is Adam; the initial learning rate is lr = 0.0001; betas = (0.937, 0.999); and weight decay = 5 × 10^−5^.

### 4.1. Simulation Results Analysis

Signal parameters of the simulation dataset: Modulation types are 16QAM, 32QAM, 64QAM, BPSK, QPSK, 8PSK, 16APSK, 32APSK, 64APSK, AM-DSB, AM-SSB, and FM; the symbol rate is 100kBaud; the carrier frequency is 350 kHz; the factor of oversampling is 10; the roll-off coefficient of the shape filter is 0.35, and time delay is 3; the SNR is -10:2:18dB; a total of 2200 signal samples are generated for each modulation type under each SNR, and the data format of each I/Q signal sample is 2 × 1000; the ratio of the number of training set and test set is 10:1.

**Figure 5:**
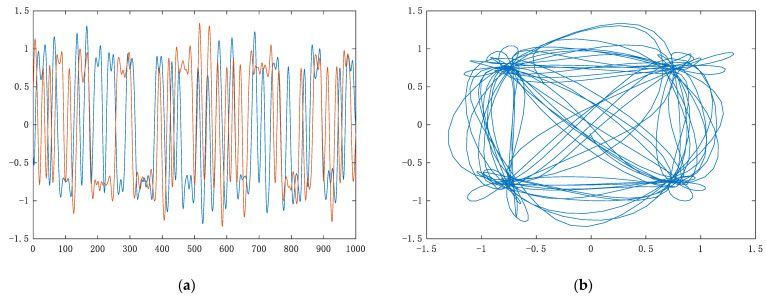

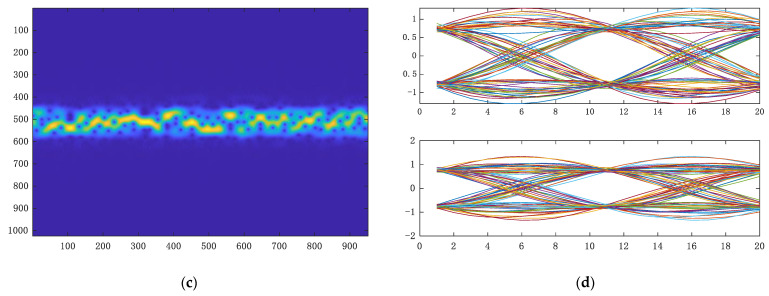
Different feature inputs (QPSK as example). (**a**) I/Q waveform; (**b**) vector diagram; (**c**) time-frequency diagram; (**d**) eye diagram.

At present, deep learning is widely used in modulation recognition. In terms of feature input, it can be generally divided into two types: One is to directly input signal data into neural networks for recognition; the other is to transform I/Q waveforms into other domain features by means of domain transformation, and finally, input them into the network for training in the form of images. According to our survey, there are relatively few studies on the systematic comparison of these two types of inputs. Hence, four common feature inputs, such as the I/Q waveform, vector diagram, eye diagram, and time-frequency diagram, are selected for preliminary research on the impact of recognition performance, as shown in Figure 5.

**Figure 6:**
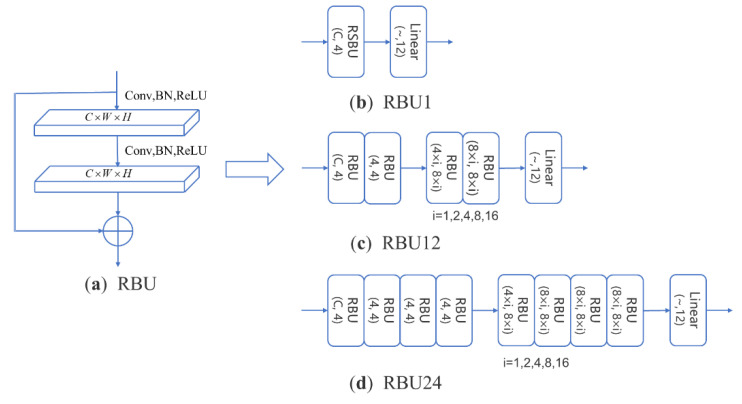
Residual network model. (**a**) RBU; (**b**) RBU1; (**c**) RBU12; and (**d**) RBU24.

To better compare the influence of different feature inputs, residual building units (RBUs) were adopted as the basic module to design three residual network models based on the ResNets structure, as shown in Figure 6. Since the data format of the I/Q waveform is 2 × 1000, the network convolution kernel is 1 × 3 and 2 × 3; the image format of the vector diagram, eye diagram, and time-frequency diagram is 224 × 224, so the network convolution kernel is 3 × 3.

Table 1 shows the overall recognition accuracy of the different feature inputs, and Table 2 shows the complexity comparison of the different network models. According to the experiment results in Table 1, it can be concluded that the I/Q waveform as a network input has the highest recognition accuracy, so the I/Q waveform serves as input to the neural network in subsequent experiments. According to our preliminary analysis, when the I/Q waveform is taken as input, the network can directly extract features from raw signal data. However, when the received signals are converted into other domain features and input in the form of images, network captures features from data distribution of images, which inevitably leads to the loss of information. Comparing three residual network models combined with Table 1 and Table 2, the recognition effect of RBU1 is not ideal. RBU24 has the highest recognition accuracy, but has numerous parameters and floating-point operations per second (FLOPs). The recognition accuracy of RBU12 is very close to that of RBU24, and the number of parameters and FLOPs are relatively smaller.

Compared with Figure 2b and Figure 6c, RSBU-CW12 is RBU12 an added sub-neural network with soft thresholding. Simultaneously, edge filling is carried out before each convolution to keep boundary information. I/Q waveform serves as network input to compare the performance of RSBU-CW12 with several other common modulation recognition network models, as shown in Table 3. As can be seen from Table 3, recognition performance of RSBU-CW12 is better than that of other network models. Compared with CLDNN(Bi-LSTM), which ranks second in overall recognition accuracy, recognition performance of RSBU-CW12 is increased by 3.62%.

**Figure 7:**
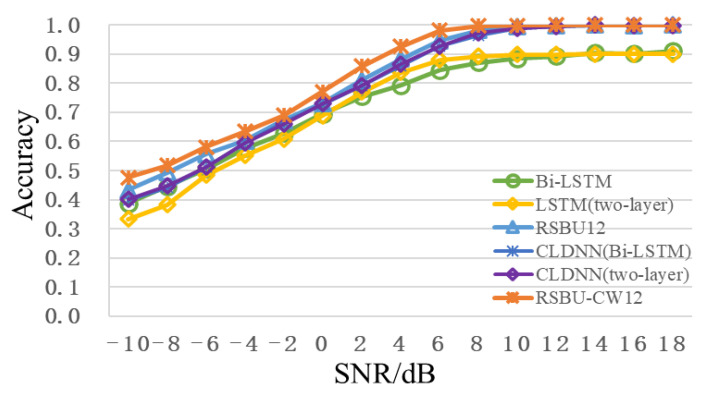
Recognition accuracy curve of different network models with change of SNR.

Figure 7 shows the recognition accuracy curve of different network models with the change of SNR. It can be seen that the recognition accuracy of RSBU-CW12 is higher than that of other network models when SNR is from -10dB to 18dB. When SNR is 2dB, the recognition accuracy of RSBU-CW12 is more than 85%, while that of other network models is less than 80%. When SNR exceeds 8dB, recognition the accuracy is approximately 100%. Advantages of RSBU-CW12 network model in modulation recognition are further illustrated by analysis, which can be used as the basic feature extraction network in subsequent research.

**Figure 8:**
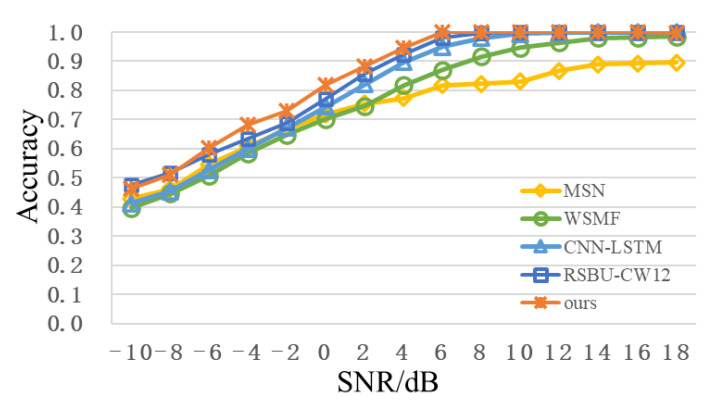
Recognition accuracy curve of different schemes with change of SNR.

To further enhance modulation recognition performance, multimodal feature fusion methods in Section 3.2 are adopted and compared with existing feature fusion schemes [14,15,16], as shown in Table 4. From Figure 8, after adding feature-fusion methods, compared with RSBU-CW12, recognition accuracy of low SNR is improved to some extent. When SNR is 0dB, the recognition accuracy is more than 80%. When SNR is 2 dB, recognition the accuracy reaches approximately 88%. When SNR is over 6dB, the recognition accuracy is approximately 100%. Meanwhile, recognition performance of feature-fusion scheme proposed in this paper is better than other feature fusion schemes. The recognition accuracy of the RSBU-CW12 is higher than those of the existing feature-fusion schemes, which indicates that RSBU-CW12 can extract more critical features. A PNN model can better integrate multimodal features to enhance recognition performance.

**Figure 9:**
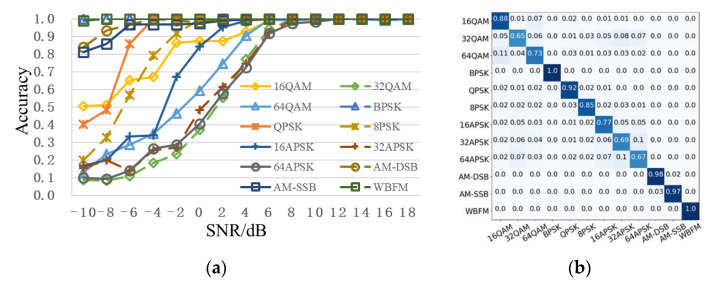
Recognition performance of the proposed scheme. (**a**) Recognition accuracy curve of each modulation type; (**b**) overall confusion matrix. The darker the color, the higher the value.

Figure 9 gives the recognition performance of the proposed scheme. Figure 9a shows the recognition accuracy curve of each modulation type. High-order modulation signals, such as 32QAM, 64QAM, 16APSK, 32APSK, and 64APSK, are very difficult to be recognized in the case of low SNR. When SNR is 6dB, recognition accuracy of all modulation types is more than 90%. Figure 9b shows the overall confusion matrix. The overall recognition accuracy of low-order modulation signals BPSK, QPSK, and analog modulation signals AM-DSB, AM-SSB, and FM is over 90% and close to 100%. The modulation order of QAM and APSK signals is higher than 16, and recognition accuracy is relatively low. Recognition accuracies of QAM, PSK, and APSK signals decrease with the increase of modulation order.

In the actual signal propagation process, signals are not only be affected by Gaussian white noise, but also face the interference of multipath fading. Therefore, Rayleigh fading and Rician fading are, respectively, added to the simulation dataset, and specific simulation channel parameters are listed in Table 5.

**Figure 10:**
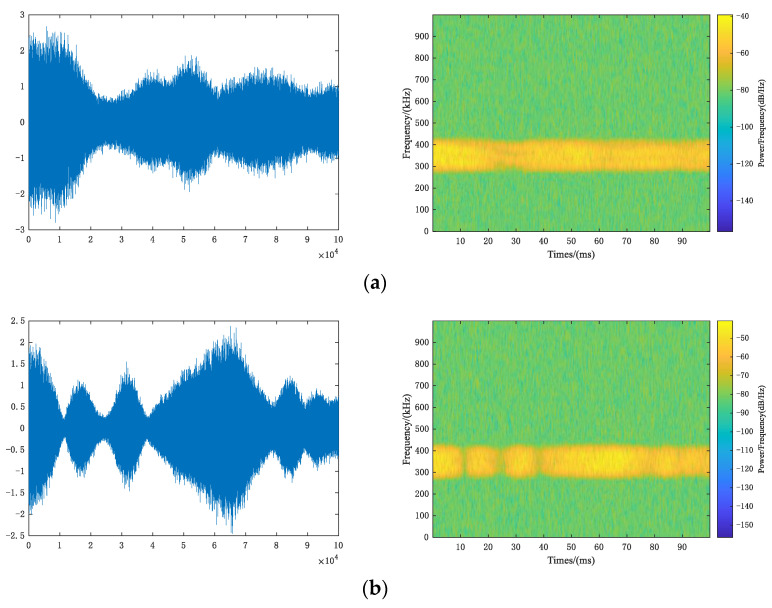
Multipath fading channel. (**a**) Rayleigh fading channel; (**b**) Rician fading channel.

Figure 10 shows the time-domain waveform (left) and the time-frequency spectrum (right) of QPSK. Through multipath fading channels, time-domain waveform becomes no longer flat. Coherent bandwidth of Rayleigh fading channel is approximately equal to 5 × 10^4^ Hz, far less than signal bandwidth 100 kHz, so frequency selective fading occurs. Coherent bandwidth of the Rician fading channel is approximately equal to 2 × 10^6^ Hz, which is greater than the signal bandwidth and belongs to flat fading; doppler frequency shift of two channels is much less than symbol rate, so both channels belong to slow fading.

**Figure 11:**
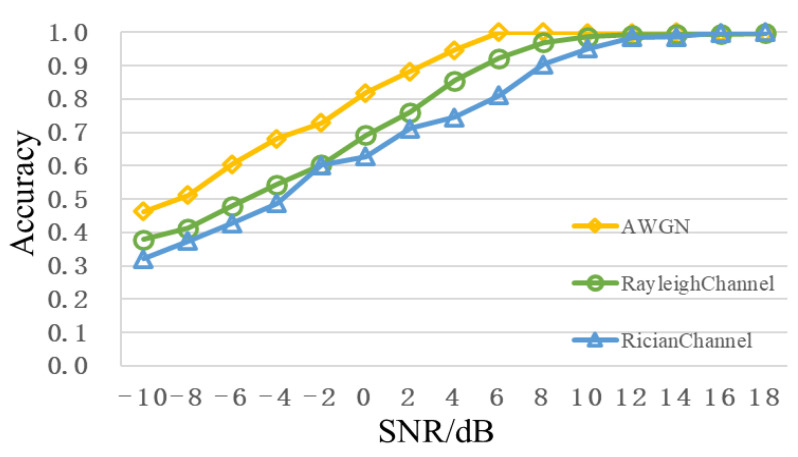
Comparison of different channel recognition performance.

Figure 11 shows the comparison of recognition performance of different channels, from which it can be seen that the Rayleigh fading and Rician fading cause different degrees of performance degradation, especially in the case of low SNR. When SNR is 0dB, recognition accuracies of the Rayleigh fading and Rician fading decrease to less than 70%. However, when SNR is greater than 8dB, the recognition accuracy can still reach more than 90%.

### 4.2. Public Dataset Validation

To further verify the performance of the proposed scheme, public dataset, RadioML2018.01A [10] is used for testing, whose parameters are shown in Table 6.

**Figure 12:**
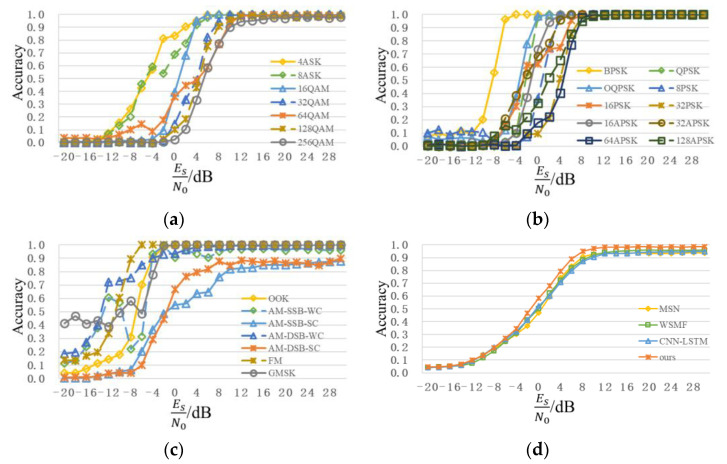
Recognition performance curve of public dataset, RadioML2018.01A. (**a**) ASK+QAM; (**b**) PSK+APSK; (**c**) low Order+Analog; (**d**) comparison of different schemes recognition performance.

Figure 12 shows a recognition performance curve of the proposed scheme in the public dataset, RadioML2018.01A. To facilitate observation, the recognition results of all the modulation types are divided into ASK+QAM, PSK+APSK, and low order+analog in Figure 12a–c. Similar to the analysis results in Figure 9, compared with low-order modulation signals, such as OOK and BPSK, recognition of the high-order modulation signals, such as 128APSK and 256QAM is more difficult and its accuracy is relatively lower. When SNR is 4dB, except for 16PSK (75%), recognition accuracies of other digital modulation signals of order 16 or less are over 90%, and recognition accuracies of OOK, BPSK, QPSK, 8PSK, and 16APSK are close to 100%. When SNR is 10dB, except for AM-DSB-SC (81.84%) and AM-SSB-SC (84.77%), recognition accuracies of other signals are more than 90%, and recognition accuracies of most signals can reach 100%; the recognition accuracy of 128APSK is 98.63%; the recognition accuracy of 128QAM is 95.70%; the recognition accuracy of 256QAM is 90.04%. For analog modulation signals, the overall recognition accuracies of FM, AM-DSB-WC, and AM-SSB-WC are high, while the highest recognition accuracies of AM-DSB-SC and AM-SSB-SC are only 87.50% and 89.84%, respectively. Figure 12d shows the overall recognition accuracy, from which it can be seen that the proposed scheme can achieve better performance than MSN, WSMF, and CNN-LSTM in the public dataset, RadioML2018.01A. With continuous improvements in SNR, the recognition accuracy also increases. When SNR is 4dB, the overall recognition accuracy is 80.22%. When SNR reaches 8dB, the overall recognition accuracy exceeds 95%, which further demonstrates the superiority of the proposed scheme model.

## 5. Conclusions

This paper proposes a modulation recognition scheme based on multimodal feature fusion to improve performance on modulation recognition under different channel interferences. Firstly, the recognition performance of the waveform data as network input is higher than that of other domain transformation features through experiment comparison, so the I/Q waveform is adopted as network input. To make more use of the useful information in the received signals, two groups of the time-frequency domain features, such as modulus and phase, welch spectrum, square spectrum, and fourth power spectrum, are extracted and fed into the network together with the I/Q waveform. The designed network RSBU-CW12 is used for spatial feature extraction, and the LSTM network is used for temporal feature extraction. The temporal and spatial features were paired with each other to increase feature diversity. The features extracted from the different inputs are further cross-fused with a PNN model, so as to enhance recognition performance.

Compared with the existing modulation recognition feature fusion schemes, the proposed scheme in this paper can effectively improve the performance of the modulation recognition. Under the condition of a multipath fading channel, performance is degraded, but the recognition effect is still good. In addition, experiment results in the public dataset, RadioML2018.01A, show that when SNR is 4dB, the overall recognition accuracy is 80.22%; when SNR reaches 8dB, the recognition accuracy can exceed 95%, which further illustrates superiority of the proposed scheme.

## Figures and Tables

**Table 1:** Overall recognition accuracy of different feature inputs.

	I/Q Waveform	Vector Diagram	Time-Frequency Diagram	Eye Diagram
RBU1	0.6526	0.6491	0.5625	0.3333
RBU12	0.7738	0.6781	0.7248	0.3488
RBU24	0.7836	0.6950	0.7426	0.3505

**Table 2:** Complexity comparison of different network models.

	RBU1	RBU12	RBU24
Parameters(M)	4.3652	2.3646	2.8997
FLOPs(M)	4.4943	9.6636	16.7901

**Table 3:** Performance of modulation recognition network models.

	Overall Recognition Accuracy
LSTM (two-layer)	0.7303
Bi-LSTM	0.7357
RSBU12	0.7738
CLDNN(LSTM)	0.7931
CLDNN(Bi-LSTM)	0.7945
RSBU-CW12	0.8307

Notes: LSTM (two-layer) represents a two-layer LSTM; CLDNN (LSTM) is a network composed of CNN, LSTM, and DNN [21]; CLDNN(Bi-LSTM) is to replace LSTM with Bi-LSTM.

**Table 4:** Comparison of feature fusion schemes.

	Feature Input	Network	Feature Fusion Method
MSN [14]	I/Q waveform	MPN	Multi-scale feature maps merging
WSMF [15]	I/Q waveform, modulus and phase, welch spectrum, square spectrum and fourth power spectrum	Resnet	Multimodal information from multiple transformation domain concatenation
CNN-LSTM [16]	I/Q waveform, modulus and phase	CNN-LSTM based dual-stream structure	The spatial-temporal feature interaction
ours	I/Q waveform, modulus and phase, welch spectrum, square spectrum and fourth power spectrum	**RSBU-CW12**, LSTM, PNN	Multimodal information from multiple transformation domain concatenation, The spatial-temporal feature interaction, **PNN Feature Cross Fusion**

**Table 5:** Specific simulation channel parameters.

Channel	Rayleigh Fading	Rician Fading
Path Delays (s)	[0.0, 2 × 10^−5^]	[0.0, 5 × 10^−7^]
Average PathGains (dB)	[0.0, −2.0]	[0.0, −2.0]
Maximum DopplerShift (Hz)	30.0	50.0
DopplerSpectrum	doppler (‘Gaussian’, 0.6)	doppler (‘Gaussian’, 0.6)
K-Factor	--	2.8
DirectPath DopplerShift	--	5.0
DirectPath InitialPhase	--	0.5

**Table 6:** Dataset parameter settings.

Dataset	RadioML2018.01A
Modulation Type (24 kinds)	OOK, 4ASK, 8ASK, BPSK, QPSK, 8PSK, 16PSK, 32PSK, 16APSK, 32APSK, 64APSK, 128APSK, 16QAM, 32QAM, 64QAM, 128QAM, 256QAM, AM-SSB-WC, AM-SSB-SC, AM-DSB-WC, AM-DSB-SC, FM, GMSK, OQPSK
*E_s_*/*N*_0_	−20:2:30 dB
Data Format	2 × 1024
Propagation Channel	Gaussian white noise, multipath fading, carrier frequency offset, delay spread, etc.
